# Disseminated tuberculosis complicated by ARDS in a third-trimester healthcare worker

**DOI:** 10.1093/omcr/omaf257

**Published:** 2025-12-26

**Authors:** Vinod Xavier, Nishma Monteiro, Renji Jos, Ge Vivin Vinister

**Affiliations:** Department of Internal Medicine, Aster Medcity, Cheranalloor, Ernakulam District, Kochi, Kerala 682027, India; Department of Anaesthesiology, Lisie Hospital, Lisie Medical and Educational Institutions, Ernakulam North, Kochi, Ernakulam District, Kerala 682018, India; Department of Internal Medicine, Aster Medcity, Cheranalloor, Ernakulam District, Kochi, Kerala 682027, India; Department of Internal Medicine, MedStar Union Memorial Hospital, Baltimore, MD 21218, United States

**Keywords:** Miliary tuberculosis, pregnancy, ARDS, placental pathology, choroidal tubercles

## Abstract

Miliary tuberculosis (TB) in pregnancy presents unique diagnostic and therapeutic challenges due to overlapping physiological changes, imaging limitations, and fetal considerations. We describe a 42-year-old third-trimester healthcare worker who presented with prolonged fever and rapidly progressive hypoxaemia. Initial investigations were inconclusive, and imaging showed subtle reticulonodular opacities. Her condition worsened into severe ARDS, requiring emergency caesarean delivery and mechanical ventilation. Placental histopathology revealed necrotizing granulomatous inflammation, and fundoscopy identified choroidal tubercles. *Mycobacterium tuberculosis* was later confirmed on bronchoalveolar lavage. Anti-tubercular therapy and corticosteroids led to gradual clinical recovery and successful extubation. The neonate remained well on follow-up. This case underscores the diagnostic complexity of miliary TB in pregnancy and the value of placental pathology and fundoscopy when conventional tools fall short. With timely intervention and multidisciplinary care, even fulminant presentations can result in favourable maternal and neonatal outcomes.

## Introduction

Miliary tuberculosis (TB) is a potentially fatal form of disseminated TB that requires early recognition and treatment to improve outcomes. Diagnosis is often delayed due to nonspecific clinical features and subtle or absent radiological findings. Diagnostic confirmation may require fundoscopy, histopathology, culture, or molecular methods [1].

Risk factors for miliary TB include diabetes, alcohol use, smoking, and HIV. Though pregnancy does not inherently worsen TB, the disease is rare and diagnostically elusive during gestation, usually linked to maternal comorbidities [2]. The combination of miliary TB and acute respiratory distress syndrome (ARDS) in pregnancy is extremely rare [3–5].

We present a case of miliary TB with ARDS in a third-trimester healthcare worker, managed successfully with multidisciplinary intervention.

## Case report

A 42-year-old South Asian staff nurse (G3P3L2) from Kuwait, at 35 weeks of gestation, presented with a one-month history of intermittent high-grade fever. She had recovered from mild COVID-19 four months earlier and had no comorbidities. Initial workup in Kuwait was inconclusive. She was referred to our center in India for further evaluation.

**Figure 1:**
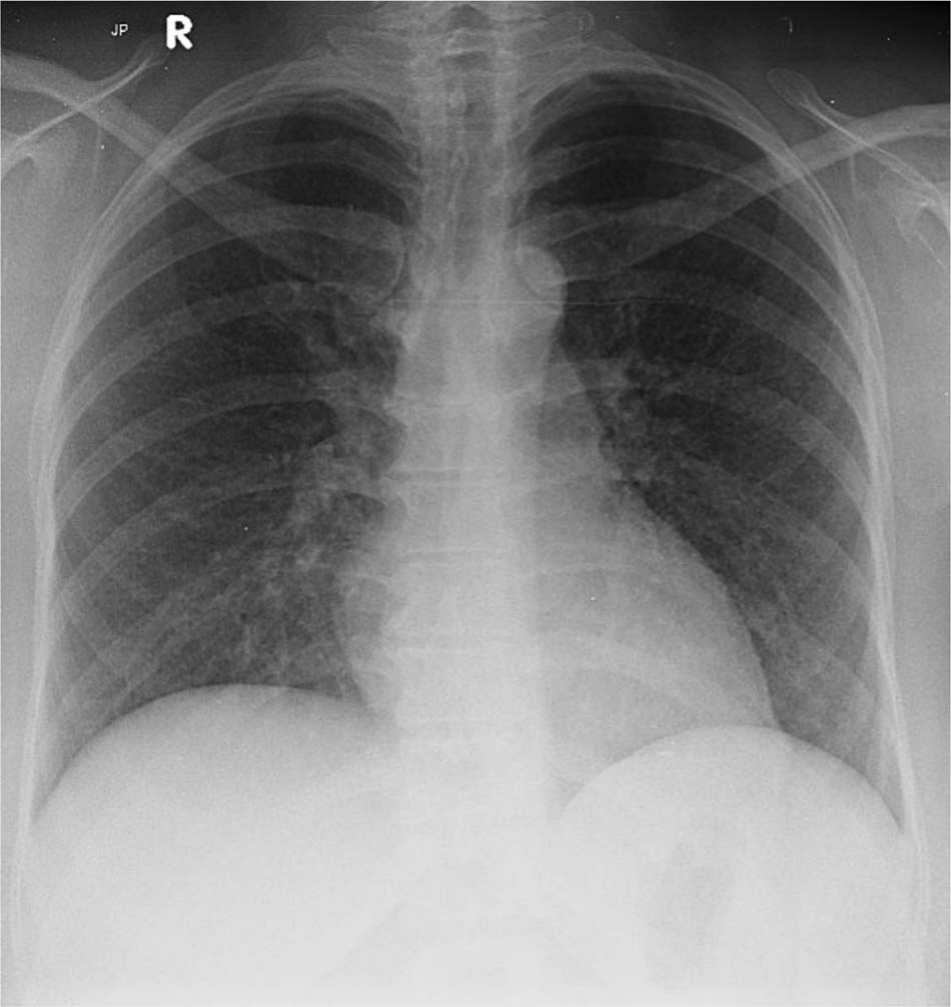
Chest radiograph showing diffuse bilateral reticulonodular opacities.

She denied night sweats or localizing symptoms, and physical examination was unremarkable. Labs revealed mild transaminitis (AST 68 U/L, ALP 480 U/L), leukocytes 6800/μL (77% neutrophils), CRP 60.8 mg/L, ESR 85 mm/h, and D-dimer > 1000 ng/mL. Chest radiograph showed a retrocardiac opacity (Fig. 1). Extensive infectious and autoimmune evaluations were negative, including AFB smear, CBNAAT, Mantoux, IGRA, and bone marrow aspiration. Ultrasound confirmed a 35 + 1 week pregnancy with mild hepatomegaly.

**Figure 2:**
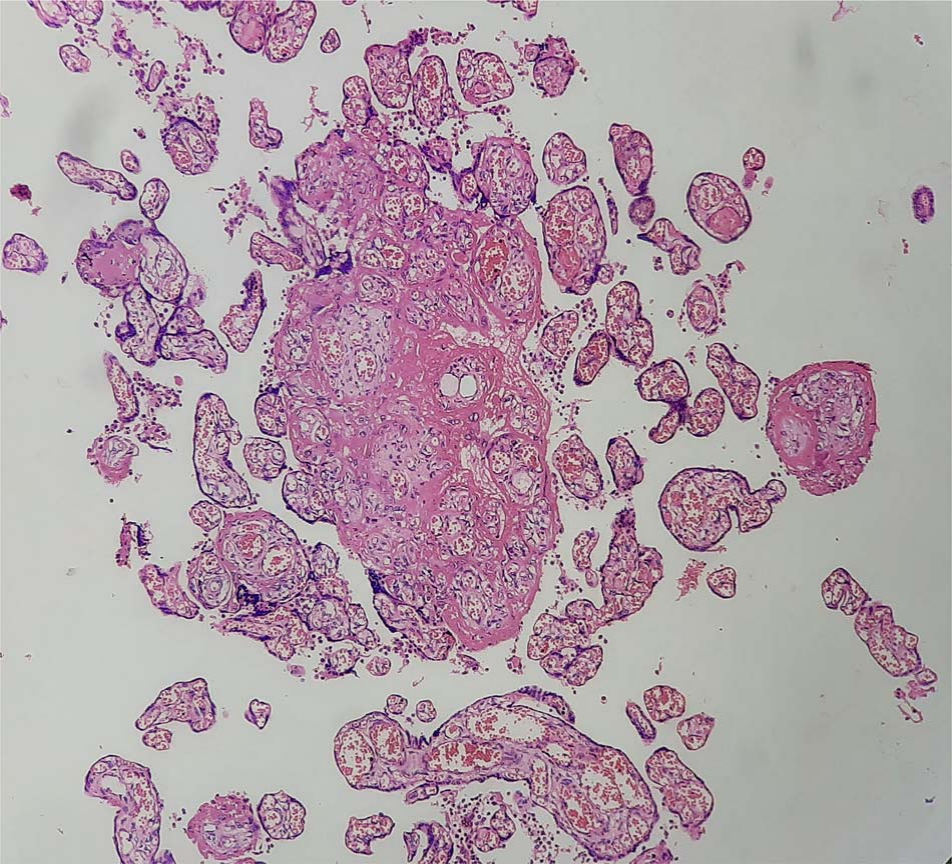
Placental villi with villitis and intervillositis.

**Figure 3:**
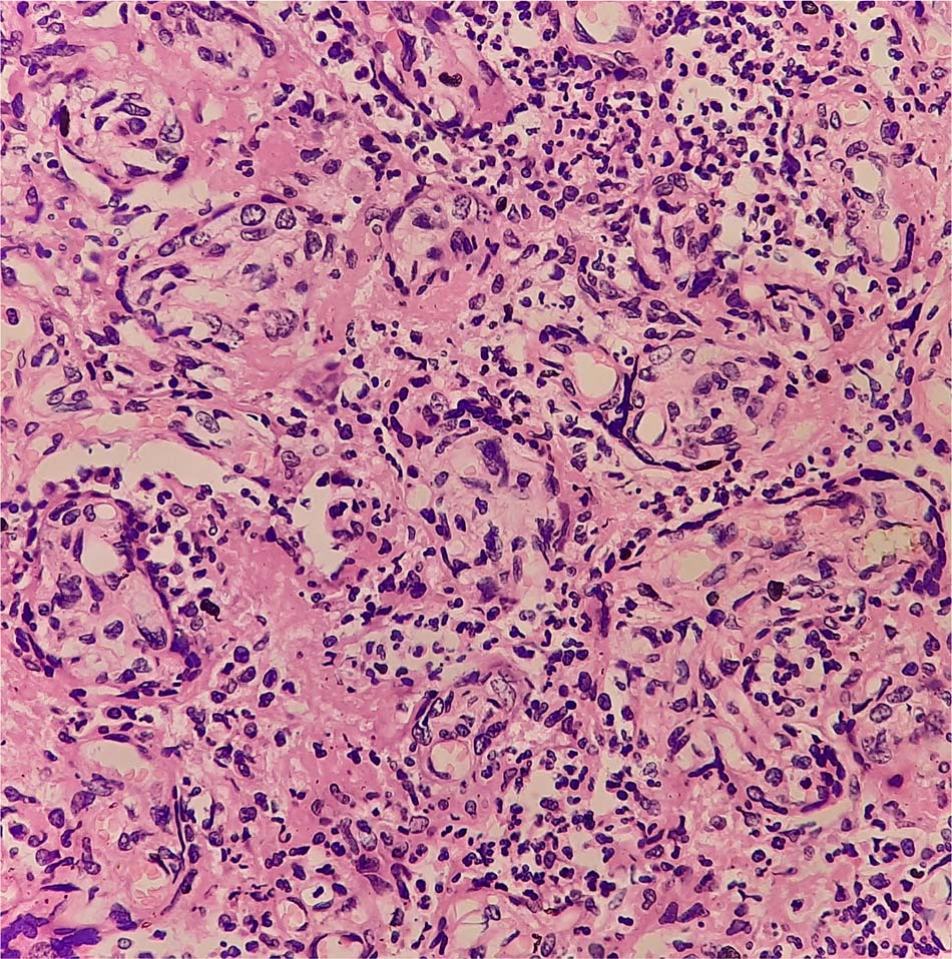
High-power view showing neutrophilic infiltrates.

**Figure 4:**
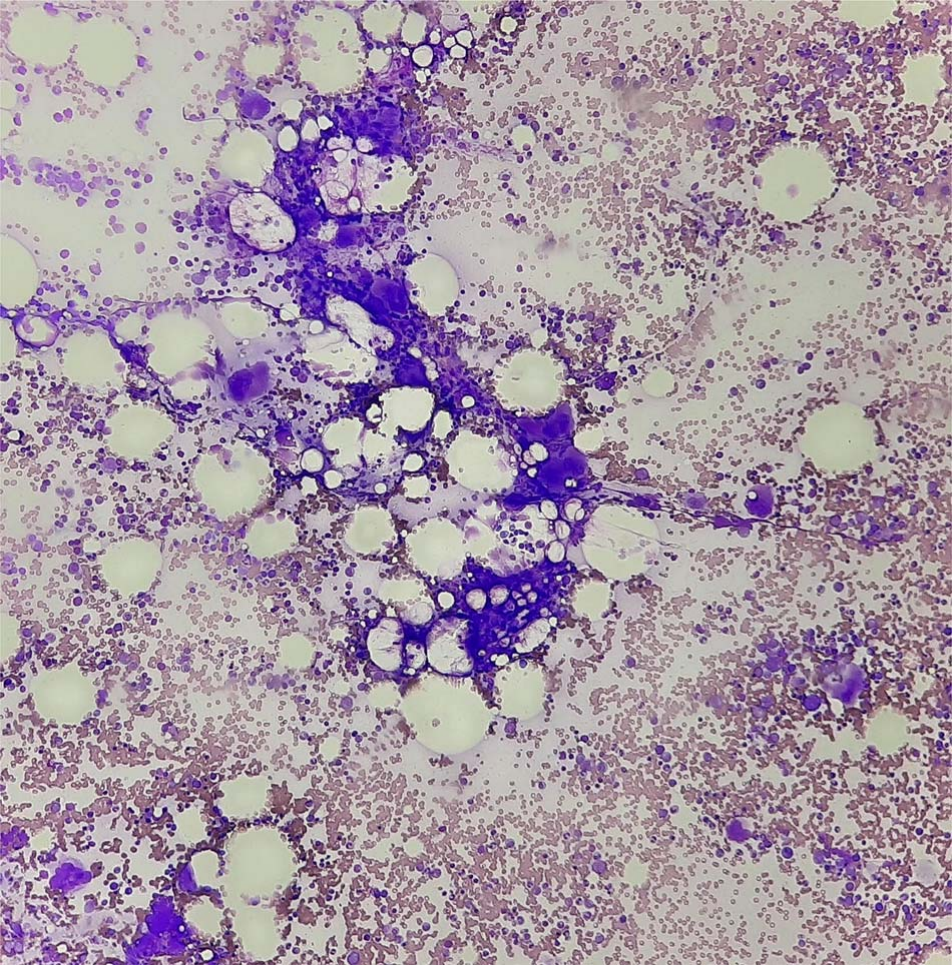
Giemsa stain with neutrophils and vacuolated histiocytes.

**Figure 5:**
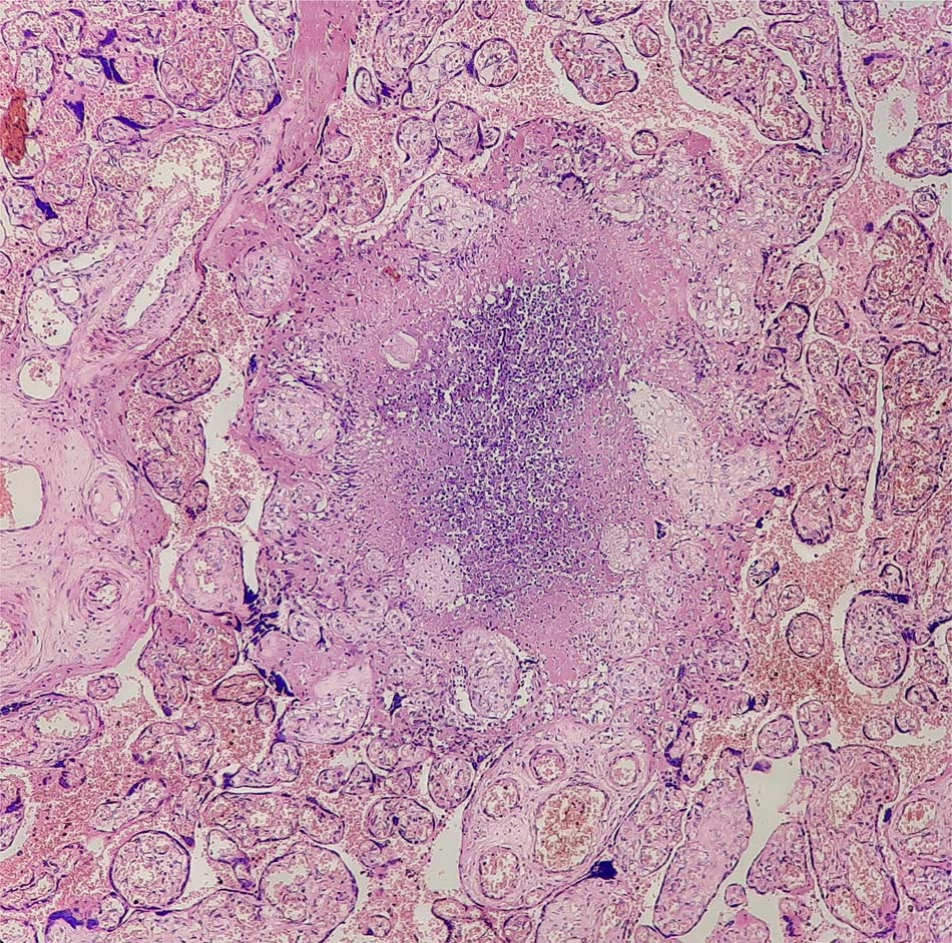
Necrotizing granulomas in placenta.

**Figure 6:**
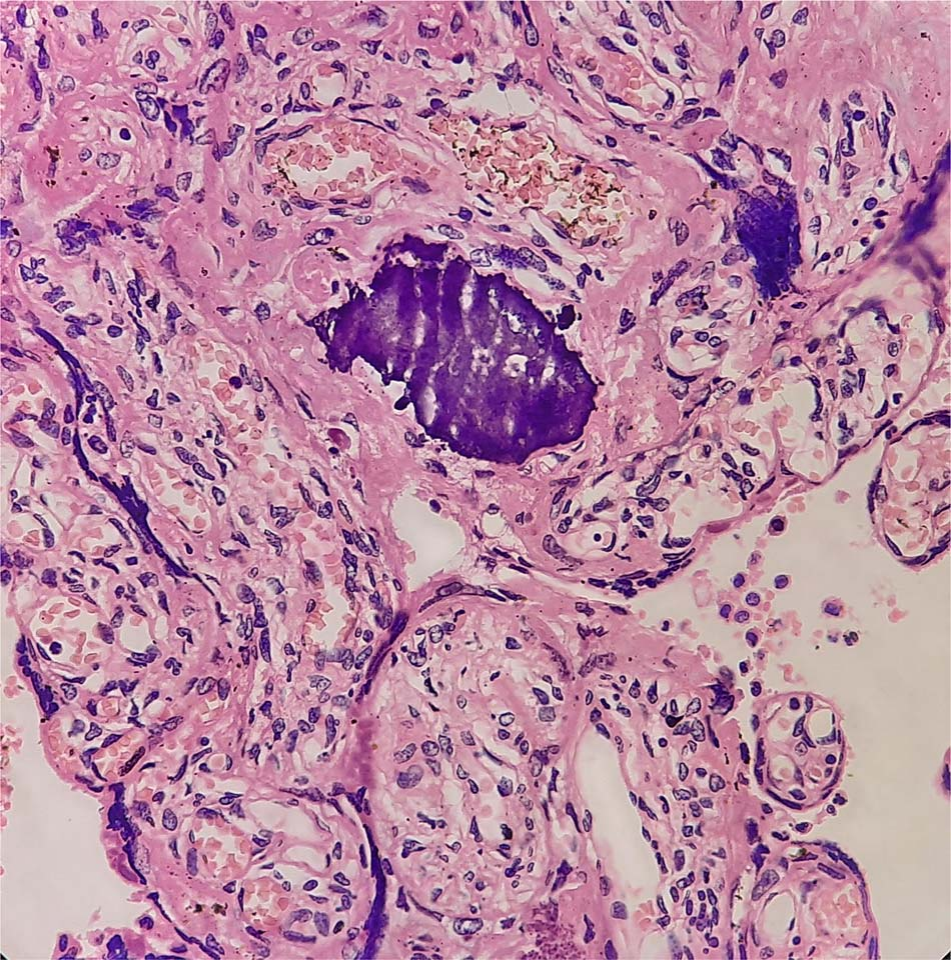
Langhans-type giant cells with caseous necrosis.

**Figure 7:**
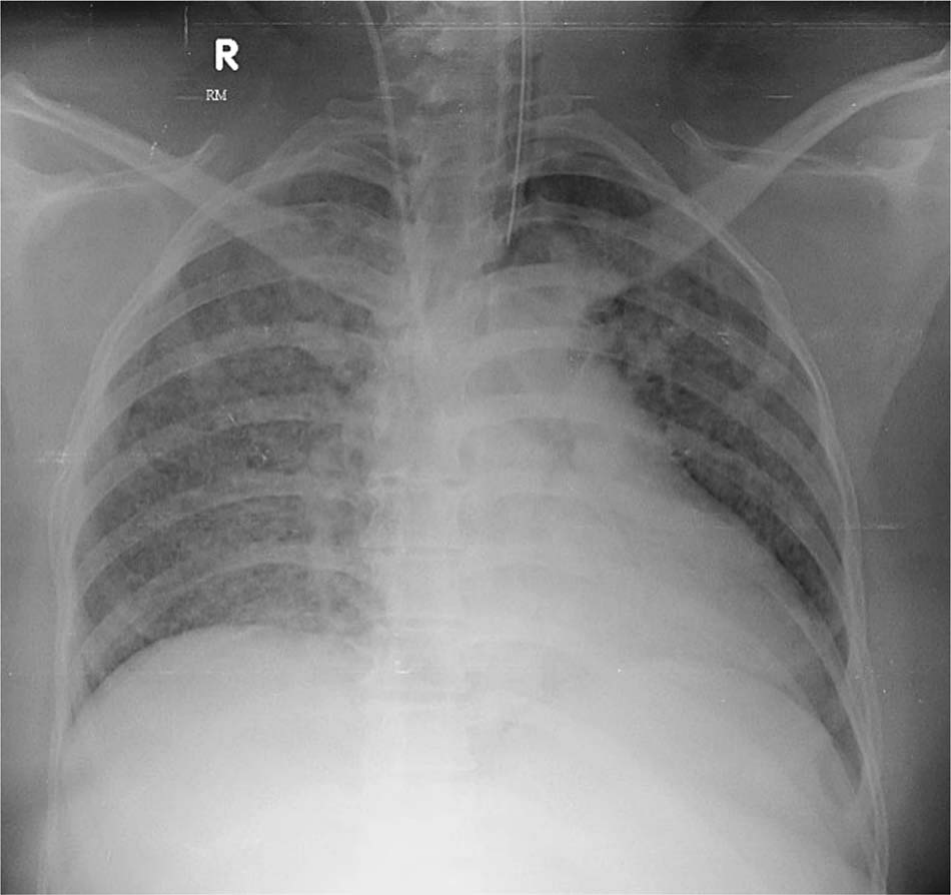
Chest radiograph showing progressive miliary mottling.

Antibiotics were paused, but fever and hypoxia worsened. She was started on Meropenem and Azithromycin. Crepitations developed, but repeat COVID PCR and sputum tests remained negative. Oxygen needs escalated. CT was declined due to fetal concerns; MRI was attempted but intolerable. During induction of labour, she developed respiratory failure, requiring intubation and emergency caesarean. A 2.42 kg neonate was delivered with low cord pH and NICU admission. She received IV methylprednisolone for ARDS. Prone ventilation was avoided post-operatively. Fever abated post-delivery. Placental histology showed villitis, intervillositis, necrosis, and granulomas (Figs 2–6). Chest radiographs progressed to miliary mottling (Fig. 7). Fundoscopy revealed choroidal tubercles. BAL was initially negative but smear became positive 48 hours after ATT initiation. CBNAAT confirmed *Mycobacterium tuberculosis* with no rifampicin resistance. She was started on HRZE and pyridoxine.

**Figure 8:**
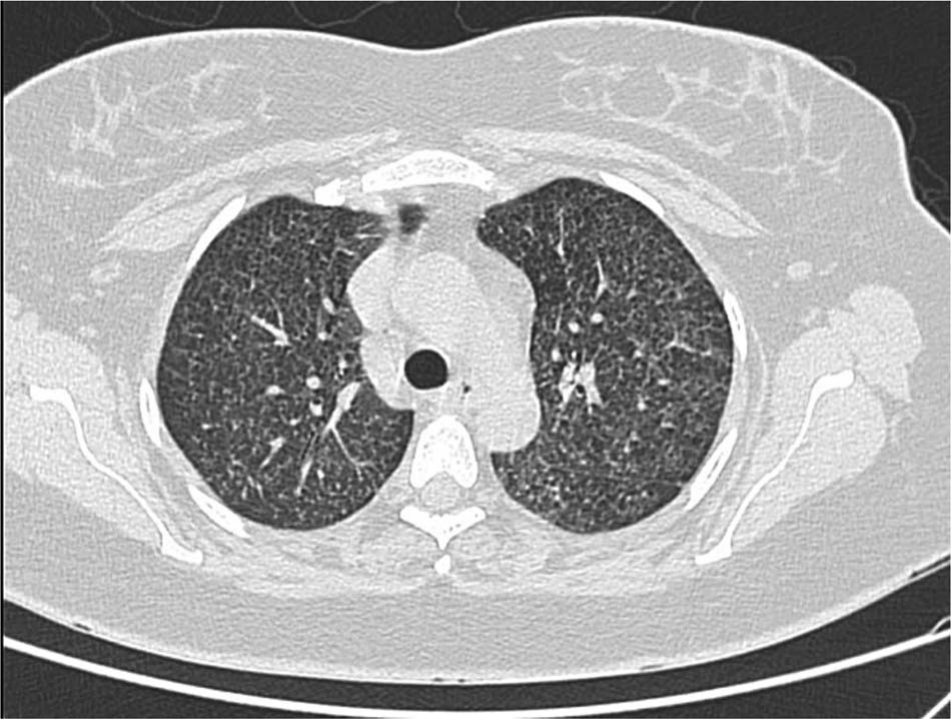
HRCT showing centrilobular nodules and ‘tree-in-bud’ opacities.

She improved and was extubated on post-op day 8. Steroids were tapered over 3 weeks. CT thorax on day 20 showed miliary nodules and segmental coalescence (Fig. 8). Complications included ventilator-associated pneumonia (*Acinetobacter baumannii*), drug-induced liver injury, post-op bleeding, and DVT involving multiple veins. ATT was briefly modified and resumed after hepatic recovery. She was discharged after 31 days. At follow-up, she was afebrile with good function. The neonate received isoniazid, rifampicin, and pyridoxine and remained well.

## Discussion

Miliary tuberculosis remains a diagnostic challenge because of its nonspecific systemic manifestations and often subtle or absent radiographic findings [1]. In pregnancy, physiological changes and restrictions on imaging add further complexity, which may delay recognition [2]. In our patient, the absence of classic respiratory symptoms, negative immunological and microbiological tests, and reluctance to pursue CT due to fetal concerns all contributed to diagnostic uncertainty.

Fundoscopic identification of choroidal tubercles, considered virtually pathognomonic, and placental histopathology showing necrotizing granulomas provided critical early clues. Similar reliance on histology or ocular findings has been described when conventional diagnostics are inconclusive [1].

The progression to ARDS in disseminated TB is rare but recognized, with high reported mortality [3]. Recent case reports describe similar presentations in pregnancy, including a 22-year-old woman with miliary TB and ARDS at 23 weeks who required ventilatory support but delivered a healthy child [4], and a 36-year-old patient in whom blood purification was used as adjunct therapy during severe ARDS [5]. The overlap with recent or preceding COVID-19 infection has also been implicated in dysregulated immunity, with a reported third-trimester case of post-COVID miliary TB complicated by ARDS [6], paralleling our patient’s prior infection history.

Pregnancy further complicates ventilatory management and limits the use of prone positioning. Corticosteroids, though not universally established in TB, may have contributed to recovery in this setting. Current guidelines, including the CDC 2025 update and the ATS/CDC/ERS/IDSA 2025 regimen update, emphasize immediate treatment initiation for active TB in pregnancy and highlight newer shortened regimens for drug-susceptible TB [7]. Although these regimens are not yet validated for miliary TB or pregnancy, they represent an evolving landscape of TB care that may influence future management.

## Consent

Written informed consent was obtained from the patient for publication.

## Guarantor

Dr. Vinod Xavier.
